# Big data analyses and individual health profiling in the arena of rheumatic and musculoskeletal diseases (RMDs)

**DOI:** 10.1177/1759720X221105978

**Published:** 2022-06-30

**Authors:** Diederik De Cock, Elena Myasoedova, Daniel Aletaha, Paul Studenic

**Affiliations:** Clinical and Experimental Endocrinology, Department of Chronic Diseases and Metabolism, KU Leuven, Leuven, Belgium; Skeletal Biology and Engineering Research Centre, KU Leuven, Leuven, Belgium; Division of Rheumatology, Department of Internal Medicine and Division of Epidemiology, Department of Quantitative Health Sciences, Mayo Clinic, Rochester, MN, USA; Division of Rheumatology, Department of Internal Medicine 3, Medical University Vienna, Vienna, Austria; Division of Rheumatology, Department of Internal Medicine 3, Medical University Vienna, Waehringer Guertel 18-20, 1090 Vienna, Austria; Division of Rheumatology, Department of Medicine (Solna), Karolinska Institutet, Stockholm, Sweden

**Keywords:** e-health, electronic health records, health care services, personalized medicine, precision medicine, precision medicine diagnostic tests, remote monitoring, rheumatic and musculoskeletal disease

## Abstract

Health care processes are under constant development and will need to embrace advances in technology and health science aiming to provide optimal care. Considering the perspective of increasing treatment options for people with rheumatic and musculoskeletal diseases, but in many cases not reaching all treatment targets that matter to patients, care systems bare potential to improve on a holistic level. This review provides an overview of systems and technologies under evaluation over the past years that show potential to impact diagnosis and treatment of rheumatic diseases in about 10 years from now. We summarize initiatives and studies from the field of electronic health records, biobanking, remote monitoring, and artificial intelligence. The combination and implementation of these opportunities in daily clinical care will be key for a new era in care of our patients. This aims to inform rheumatologists and healthcare providers concerned with chronic inflammatory musculoskeletal conditions about current important and promising developments in science that might substantially impact the management processes of rheumatic diseases in the 2030s.

## Introduction

Rheumatic and musculoskeletal diseases (RMDs) are a major threat to quality of life and affect a significant number of people.^1–3^ RMDs are often divided in two groups, that is, inflammatory diseases and degenerative disorders, with the latter ones being more prevalent. On population level, RMDs coincide with the highest rates of years lived with disability: 6.6% of disability adjusted life years are attributable to them in Europe, 4.6% in America, and 4.2% in Asia.^4^ Most rheumatologists primarily manage inflammatory rheumatic conditions. Rheumatoid arthritis (RA) is the most prevalent inflammatory arthritis, with a worldwide prevalence of 0.5%–1%.^3,5^ Treatment options have tremendously improved over the past decades. Patients 20 years ago often had to fear long-term adverse outcome such as a loss of their ability to work due to insufficient disease control because of a lack therapeutic options, and due to the resulting disability.^6^ The first decade of the XXI century brought many advanced therapeutic options with it, including biologic disease modifying antirheumatic drugs (bDMARDs). The armamentarium of bDMARDs was expanded in the second decade and medications have been made more accessible to patients with addition of biosimilars and the coverage of costs by the public health system in Europe.^7–9^ Over the past years, following therapeutic advancements and introduction of treat-to-target concept, we have achieved significant improvement in disease activity control on the population level, particularly in RA. However, not all patients achieve optimal control of their disease activity and individualized approach to treatment of rheumatic diseases remains elusive.^10–12^ Which mechanisms can we modify to improve outcomes and in particular quality of life and workability of patients with RMDs? In particular concerning inflammatory arthritis, many patients reach the official targets of low disease activity or remission, still many patients do not achieve targets based on patient-reported outcomes (PROs).^13^ Again, at the moment, we have no ability to assess which pharmacological and non-pharmacological treatment works best for those individual patients. Moreover, options for self-management strategies and comprehensive care of patients with chronic diseases in an interdisciplinary setting are beyond reach in most rheumatology clinics.^14–16^

We would need to design our care of patients more tailored to the patients’ needs, taking the socioeconomic background and multimorbidity into account.^17–19^ To many health care professionals, this call for tailored treatment is no longer novel and there is lack of supporting infrastructure that might facilitate an integrative patient-driven care process. The dilemma behind these unmet needs is unfortunately further intensified by impeding structural changes awaiting patients and health care facilities. There is an increasing shortage of health care professionals. Rheumatology care is understaffed on both, physicians and nursing staff, and the ratio of patients and care providers is far from balanced. This shortage underscores major shortcomings in the contemporary health care systems, that warrant restructuring, in order to optimize the healthcare work-flow and tailor the healthcare delivery processes to the individual patient’s needs. In the end, our systems store more data on individual patients than ever before. The advances made in imaging technologies, blood-based biomarker, connection of data in an electronic health care system are in total epitomized as big data, that in medical clinical practice has not been utilized for analyses to retrieve patient tailored solutions.^20^

This review discusses the recent technologies and innovations that are expected to benefit clinical practice in the early 2030s. It will touch on opportunities that can expedite rheumatology healthcare, make it, more efficient and highlights promising innovative research findings that will impact the diagnosis and treatment of rheumatic diseases in clinical practice in the future.

### Electronic health care records

Precision medicine, which can be defined as tailoring diagnostic, prognostic, and therapeutic strategies to each patient, have been one of the main goals of clinical research and is actively promoted by many governing bodies.^21–23^ One of the pathways to achieve precision medicine is to use the vast amounts of complex biomedical data incorporated in the electronic health records (EHRs) of clinics and similar institutions. EHR data together with ‘omics’-data are regularly called Big Data.^24^ Due to the complexity and scale of this data, many therefore interpret big data analytics to only use complex analytical techniques such as artificial intelligence and machine learning. Arguably, these techniques will aid in the analysis and interpretation of the results, yet Big Data analysis is more than just these techniques. It is better to define Big Data analysis as all the instruments one needs to interpret the data correctly. Therefore, Big Data analytics are all the statistical tools necessary to cope with the challenges of data frequency, quality, dimensionality, and heterogeneity.^25^ In succeeding so, EHRs become valuable data sources, which we will discuss in the following chapter in detail.

The practice of medical record keeping has transformed dramatically over time, especially in the last two decades. From identifying a patient`s name to detailed structured descriptions of physical exam and recommendations, medical recording constitutes an important aspect of a clinical visit.^26^ The digitalisation of EHR systems led to further advances in medical documentation. Transition from paper to electronic databases, EHRs now generally include prompts or even requirements for healthcare professionals (HCPs) to provide certain details in explicit formats. HCPs may develop ways to side-step these requirements.^27^ Inaccurate or incomplete documentation may even influence patient safety and quality of care. It has also been shown that record practices do not necessarily align with clinical need or require excessive additional clinical time.^28^

Despite academic interest in the use of EHRs, research has yet to explore the full potential of these databases in healthcare. In their digital form, EHRs offer potential beyond their original paper counterparts through (big) data analytics. Health data and big data analyses, however, do not happen independently of organizational and professional participation in the format of emerging digital recording practices. Each socio-technical context should take the social processes and decisions into account when analysing these big data. In contrast, however, a review of editorials dealing with big data in academic health journals found that more than half the editorials assumed a direct correlation between big volumes of data and knowledge or truth.^29^

Although the above-mentioned preconceptions and hesitations exist, EHRs appear to be an inevitable technological advance. Nationally linked EHR systems remain relatively rare but are being developed in a few countries around the world. A prime example of such a EHR database are the Hospital Episode Statistics (HES) data, which report on all clinical activity in generally all hospitals in the United Kingdom. These data are submitted centrally and used for funding, resource allocation, trend monitoring, and conceptualization of performance and clinical indicators. Such national EHR is appealing to look at trends in disease prevalence and healthcare usage. For example, Hannah *et al*.^30^ used HES to observe national trends in hospitalization rates in England between 1998 and 2015 for Systemic lupus erythematosus (SLE) and RA. Authors reported that while RA emergency admissions rose, those for SLE decreased. Length of stay in the hospital and bed days were reduced for both conditions. Another example is a study that investigated the trends in arthroscopic knee surgery rate in England between 1997 and 2017. This study used data from over a million surgical interventions spanning 20 years and found a vast decrease in interventions. This example outlines the possibility of using national EHR data alone, yet another appealing use is to link such databases, either to other EHR databases or to existing patient cohorts. Aside of HES, Clinical Practice Research Datalink (CPRD) is a research platform that collects anonymised patient data from a network of general practitioner (GP) practices across the United Kingdom. A study linking CPRD and HES, for example, looked at the FRAX (Fracture Risk Assessment) score both in patients with RA and in the general population.^31^ It was found that the fracture risk was overestimated in RA, but FRAX performed well for hip fracture in the general population. Some studies link EHR to existing cohorts to enrich or validate their results. Nikiphorou *et al.*^32^ used HES, for example, as a validation tool to examine predictors for length of stay for orthopaedic intervention in RA in two inception RA cohorts in the United Kingdom.

Of course, such huge national EHR are more the exception than the rule. Such endeavours ask a massive organizational effort, and most importantly, it must be possible to safely link such vast yet sensitive data to each other. In fact, one can even argue that databases as HES or CPRD are true EHR as these databases give only a limited snapshot of all data collected for a specific purpose. In contrast to such advanced databases, many EHR are only limitedly structured and possess various free-text variables. Advances in methodology using machine learning algorithms are promising to convert such unstructured data platforms into workable research databases. However, rheumatology research is in this respect only in infancy as, for example, research groups are now just developing machine learning techniques to reliably identify rheumatic diagnoses in unstructured EHR, which should of course form the base of any research topic in a specific condition.^33,34^

### Biobanks for clinical care in Rheumatology

Due to genetic polymorphism and uncontrolled variation of environmental risk factors, patients with RMD have substantial heterogeneity in disease pathogenesis, clinical presentation and treatment response, resulting in diagnostic challenges, difficult treatment choices and suboptimal effectiveness of current antirheumatic treatments.^35^ Understanding the heterogeneity of RMDs is an area of rapidly growing research and clinical interest that has been consistently highlighted as a primary unmet need in rheumatology by the Targeted Therapies Meeting Panel 2017–2019.^35–37^ Disentangling the heterogeneity of RMDs and their subtypes by enriching a comprehensive description of clinical phenotype with complex biological datasets of multiple ‘omics’, that is, genome, proteome, transcriptome, epigenome, and microbiome is a key step to improving our understanding of the pathogenesis of RMDs and developing predictive tools for therapeutic response. This approach is a premise for personalized or phenotype-specific medicine, with particular emphasis on patients with RMDs who have treatment-refractory disease as an unmet research need of high priority.^35^

Developing a robust scientific infrastructure by linking the well-characterized, longitudinal inception cohorts of patients with RMDs with biobanks and developing multi-centre collaborations supported by data-sharing platforms, standardized ethical regulations and well-developed legislative infrastructure could help assemble large patient cohorts and address the high-priority unmet research needs, in order to eventually improve disease outcomes for patients with RMDs.

Biobanking has emerged and grown as a relatively new field within the last 25 years, with currently existing large population-wide biobanks in several developed countries: for example, UK Biobank (*n* = 500,000 participants), Danish National Biobank (*n* > 800,000 participants), and many disease-specific Biobanks, including Biobanks for RMD patients: for example, Veterans Affairs Rheumatoid Arthritis (VARA) Biospecimen Bank, Rheumatoid Arthritis Investigational Network (RAIN) biobank, The National Bank for Rheumatic Diseases, Australian Arthritis, the biobank of Vienna and the Autoimmune Biobank collaborative (A3BC).^38,39^ In rheumatology among other specialties, biobanks are increasingly used for collaborative research to address vital public health questions and are essential for research in precision medicine.^38^

One of the examples of such collaborative initiatives is a Single Hub and Access point for paediatric Rheumatology in Europe (SHARE) initiative, which aims to optimize clinical care and research for children with RMDs across Europe.^40^ The SHARE initiative has developed the first European recommendations for collaborative, paediatric research including biobanking for children with RMDs which comprise a robust framework for a transformative collaborative research in paediatric RMDs across Europe. Another example of a large collaborative initiative with the focus on biomarker research for individualized diagnosing, prediction, and monitoring of RMDs has been launched in Denmark as an observational, prospective, translational research study of patients with RMDs followed in the nationwide Danish DANBIO Registry and the Danish Rheumatologic Biobank.^41^

The protocol for a randomized multicenter single-blind active controlled clinical trial (PREDIRA) is the first validation study designed as a single-blind controlled multicenter clinical trial of a bDMARD response prediction software (i.e. SinnoTest® algorithms) aiming at evaluating efficacy, safety, and cost-effectiveness of the web-based platform for clinical care of RA.^42^ A biobank for proteomic profiling will be generated as part of the study and used for the prediction, thus bringing personalized medicine into the management of RA.

The Tapestry DNA Sequencing Research Study is a large collaborative initiative between the Mayo Clinic, United States, and the Helix, a population genomics company (https://www.mayo.edu/research/centres-programmes/centre-individualized-medicine/research/clinical-studies/tapestry). This prospective study is designed to understand the short-term and long-term impact of genetic testing on people’s health care when their DNA results are part of the EHR. Apart from informing the innovative genetic-driven personalized research, this initiative also provides clinically actionable genetic findings for patients and providers, and vast collaboration opportunities, all operationalized through the Omics data platform. As part of predictive screening for generally healthy adults, 11.6% of participants tested positive for clinically actionable, likely pathogenic or pathogenic genetic variants.^43^

In summary, biobanking is essential for future global health research and development of personalized medicine in rheumatology. As biomarker discovery and biobanking in RMDs are gaining research momentum in the developed countries, there is a growing interest in connecting biobanking with clinical care in patients with RMDs, as exemplified by several large initiatives described above.

### Artificial intelligence to support clinical care

**Artificial intelligence** (AI) research (Table 1) is rapidly transforming healthcare, uncovering unparalleled capabilities of machine learning (ML) in data analysis and data management to inform individualized clinical decision making and improve patient outcomes across the spectrum of medical specialties, including rheumatology.^47–49^ The COVID-19 pandemic has precipitated the digital revolution resulting in unprecedented rise in research of information and communication technology in support of patient health. The ability of AI to efficiently process multidimensional data and uncover associations and combinations of data without researcher’s guidance is particularly valuable in complex chronic diseases, such as RMDs, due to high heterogeneity of patients‘ characteristics, and a multitude of treatment and outcome trajectories. AI methods help advance our understanding of RMD pathogenesis, risk stratification and outcome prediction, and inform novel research avenues for identification of new drug targets and options for drug repurposing in rare RMDs.^50,51^

**Table 1.** Definition of concepts in utilizing/analysing big data.^20,21,44–46^.

Big Data	The term refers, not only to the high volume of data, but also to the speed of new data generation (data influx) and the heterogeneity of data sources and storage formats. The 3 V: Volume, Velocity & Variety
Big Data analytics	Is the summary of structuring, cleaning and connecting different data sets and data models. To handle these tasks, artificial intelligence systems are increasingly employed.
Artificial Intelligence	The term is a summary of automated methods and historically relates to the question of autonomous work by machines, simulating human intelligence. It relates to automated devices that scan the environment and take decisions towards the highest chance of achieving a goal.
Machine Learning (ML)	ML is a method of artificial intelligence, learns by testing and training, and improves by that. It generally works by two different concepts.
Supervised Machine Learning	With this approach, data is split in a labelled training set and a validation set. By learning first the constellation of data labelled with the desired output, the system then tries to apply this model in the validation data set.
Unsupervised Machine Learning	In this case, no defined training set is used but data is organized and analysed by common characteristics that are identified by the systems algorithm. This is commonly used for clustering and dimension reduction.
Deep Learning	This can be regarded as a sophisticated subset of ML. Multiple layers of data representation as well as abstraction of data are connected and recognize distinct details and learn level by level until the final output layer. It is inspired by the neuronal system of the brain.

Examples of digitalization tools in rheumatology that hold promise for implementation in clinical rheumatology practice as part of individualized medicine include:

1. Patient clustering based on clinical and biologic features to identify distinct disease subsets using unsupervised ML data.^52–56^ This approach is used across the RMDs to overcome heterogeneity and uncertainty of diagnosis yielded by available criteria of RMDs and to identify homogeneous subgroups of RMD patients with similar clinical and biological features. This approach may help improve outcome prognostication and identification of the most efficacious treatments. However, since clustering is not contingent on labelled data and there is no mathematical metrics to assess model performance, evaluation, and validation of the model can be challenging which precludes interpretation of the clinical utility and clinical impact of the results.^50^
2. Algorithmic classification of patient medical data (i.e. clinical data, biometrics, medical images, laboratories, medications) using supervised ML methods. This approach can be applied to patient registry data, EHR, or biometrics data to classify patients with a certain RMD state (e.g. RA flare versus no flare) or certain RMD type (e.g. SLE, Sjogren syndrome) from other RMDs using available sociodemographic, clinical, and biomarker data, addressing the need for improved identification and classification of patients with RMDs, as well as identifying novel avenues for drug repurposing.^34,57–61^ The accuracy of available models varies from moderate-to-excellent in the training datasets. More successful studies compare different ML models to identify the best performing model.^57,60,61^ However, many studies have small number of patients and lack external validation analysis, precluding understanding of clinical applicability and implementation in clinical practice.
3. Prediction models using clinical data and multi-omics to forecast the risk of RMD development, predict disease outcomes, and response to antirheumatic treatments as a premise for individualized approach to medication selection in rheumatology.^47,62–71^ These studies are expected to inform prognosis of RMDs and to aid in improving treatment outcomes by overcoming the currently used ‘trial-and-error’ approach to treatment of RMDs. Most of these studies are hypothesis-generating and their clinical utility is uncertain. Validation of the models in large independent datasets (i.e. prospective observational cohorts or clinical trials) will be an important step prior to implementation in clinical practice. One of the examples of successful application of a deep ML algorithm to clinical data in the general population is the use of routine 12-lead ElektroCardioGram (ECG)data for prediction of depressed left ventricular function with good accuracy in routine practice (accuracy 86.5%, area under the curve 0.918).^72^ Such studies using low-cost routine clinical methods can help identify high-risk populations for more in-depth evaluation, which can save medical costs and improve patient outcomes.

#### Machine learning for patient identification from electronic health records as a premise for improved clinical decision-making and research in RMDs

The number of studies aimed at algorithmically identifying patients with RMDs from the EHR is rapidly increasing, thus bridging the gap between the big data and personalized medicine. One of the recent studies used gradient boosting methods to identify and predict difficult to treat RA in structured and unstructured routine care data, using clinically classified RA as a validation set, and achieved area under the curve (AUC) 0.88 for identification and AUC of 0.73 for prediction of difficult to treat RA.^58^ An innovative unsupervised automatic phenotyping algorithm (PheVis) combining diagnostic codes with medical record data showed excellent performance for identification of RA patients (cross-validated AUC 0.94) and can be evaluated for other chronic conditions.^73^ Another study used support vector machines to classify patients with RA using free-text EHR data validated against manual chart review applying 1987 and 2010 RA classification criteria, resulting in accurate classification of patients with RA (positive predictive value, PPV, 0.86, negative predictive value, NPV, 0.99) and enabling fast patient data extraction from the huge EHR resource.^34^ Prediction of individual risk of RA disease flares among participants of a randomized controlled trial of treatment withdrawal (RETRO) was performed using the stacking meta-classifier method with nested cross-validation and showed a promising AUC 0.81.^74^ Externally validating and operationalizing these approaches for rheumatology care and research holds premise for improving outcomes for patients with RA overall, and particularly for patients with refractory RA who do not benefit from treatments despite advanced combinations of effective therapeutics and represent the greatest unmet need in RA management in the developed world.^35^

Several studies examined classifiers for phenotyping SLE patients from EHR data.^33,57^ ML models of a high-performing algorithmic identification of patients with SLE using EHR data have been developed (PPV = 90%) and externally validated (AUC ~0.90), enabling accurate identification of SLE patients from multidimensional data for clinical and research purposes.^33^ Gradient boosting methods have been successfully applied for prediction and risk stratification of SLE renal flare using a Chinese national registry data (C-index ~0.75 in both, study derivation cohort and internal validation cohort).^71^ Prediction of 30-day hospital readmission for SLE patients using Cerner HealthFacts EHR database showed higher predictive performance of models using deep ML methods (AUC = 0.70) compared to traditional ML classification methods (AUC = 0.66), potentially due to the ability of deep ML methods to leverage the temporal changes in disease characteristics and their progression over time.^75^ One of the successful initiatives developed a ML algorithm for classifying patients with early SLE based on 14 variably weighted clinical and serological features in a discovery dataset from an SLE registry and tested its performance in an external validation cohort with an excellent discriminative performance (AUC = 0.98).^59^ In addition, this study proposed a clinician-friendly scoring system for early SLE diagnosis, serving as an example of linking the innovative research with clinical practice.

To address the clinical practice gap of incomplete documentation of disease activity scores for RA and SLE in real-world datasets, studies have applied ML methods for estimation and prediction of DAS28-ESR (AUC = 0.73) and SLEDAI measures (AUC = 0.93), using large RA and SLE registry data.^63,76^ Such endeavours enable more effective use of real-world data sources for research and can potentially support personalized care approach in clinical practice.

Growing number of studies recognize the great promise and rising potential of ML methods in revolutionizing research, disease management, and patient care for patients with RMDs.^50,77,78^ However, ML methods are still rudimentary and not ready for prime time. Indeed, implementation of the novel ML prediction models in routine rheumatology practice meets technical, methodological and ethical limitations,^77^ including small sample size (particularly in rare RMDs), lack of external validation, difficulty in operationalizing and implementing the models in independent clinical data set, and thus uncertain clinical utility.^50^ For example, ML model predicting the diagnosis of AS had accuracy of 0.81 in the training dataset, but had inferior performance in the data set not used in the original model development, yielding a PPV of only 6.24%, although this value was still higher than that of clinical model using Assessment of SpondyloArthritis international Society classification criteria (PPV = 1.29%) and higher than PPV of the logistic regression model (PPV = 2.55%).^79^ Comparing ML model performance to other ML models, manual modelling and clinical identification techniques can provide additional insight on the added benefits of using ML methods in real-world setting. A recent large study of methotrexate (MTX)-naïve patients with early RA showed that ML methods integrating baseline clinical data did not significantly improve prediction of MTX treatment persistence at 12 months compared to manual modelling, and the highest AUC for the best predicting ML model was only 0.67.^80^ Thus, external validation of ML models in large independent datasets with evaluation of clinical impact and cost-effectiveness compared to other available models are the necessary next steps in ML model development, as part of translation of clinical research to clinical practice, according to the Prognosis Research Strategy (PROGRESS) framework.^81^

### Remote monitoring

Mobile health (mHealth) is a novel way to close the gap in communication and reporting between prominent healthcare stakeholders such as patients, their families, and health care professionals. This is enabled by generating a network with mobile and specialized tools including smartphone apps or wearable sensors, to record and gather health data. Health information can subsequently be shared between patient and health care professionals to engage in a shared decision. It promises to promote further self-management of the disease by making the patient more engaged and by equipping them actively to take initiative in their health care. Likewise in regard of research purposes, mhealth seems to be a novel approach of collecting and analysing large amounts of data, using both participant input (self-report data) and the in-built functions of devices, such as Global Positioning System (GPS) tracking (passively measured data).

For musculoskeletal care, treatment strategies that have emerged the last two decades including treatment principles such as tight control and treat to target, created a relative shortage of practicing rheumatologists and other healthcare professionals due to the need of a more intensive follow-up of patients.^82^ Therefore, attention is turning towards new care models for rheumatic conditions, including the use of mHealth applications, such as mobile apps and wearables. When routinely integrated into health care and linked to electronic medical patient files, these mHealth-applications and wearables could prove to be an added value in (tele)monitoring treatment adherence, adverse treatment effects or symptoms, and disease activity in RMDs.^83^ Specifically, remote monitoring of a well-selected set of PROs and wearable-obtained physical parameters could provide continuous information on a patient’s health status.^84^ Such a remote monitoring approach holds promise to predict the need for urgent clinic visits in patients with high disease activity, but also to reduce the number of clinical visits for well-controlled patients.^85^ By consequence, routine use of a personalized remote monitoring system holds the potential to increase the quality of care for patients with RMDs, as well as being a possible cost-saving measure.^86^

However, although the hopes are high, reality shows a more distorted image of mHealth in RMD care. A systematic review by Najm *et al.*^87^ revealed a vast heterogeneity in designs, purposes and users for self-management mHealth apps in RMDs. Moreover, relevance and usage of the data collected by the apps were questionable and not always clearly defined. First, healthcare professionals were not always involved in the development of these apps. Second, the funding, origin and design processes of these tools are lacking details in many papers. Third, there seems to be a high turnover in apps, as only a minority of them is still to be commercially available. A review of apps for patients with RA available in Google Play and Itunes from 2019 did in the end only include 20 apps for further analyses of which only five were comprehensive, enabling symptom tracking, education, and management by using one tool.^88^

In parallel to this review, a EULAR initiative published points-to-consider as a guidance to develop mHealth applications.^89^ As for the use of mHealth in daily clinical care, the opportunities for mHealth in rheumatology research seemed without boundaries. One of the many hurdles rheumatology research is confronted with, is the fact that RMDs may manifest in numerous ways and that the impact of RMDs is in general reported by patients through the experience of highly fluctuating, challenging manageably and unpredictable symptoms. As classic research by default is unable to capture this kind of data, our understanding remains limited. Thus, mHealth could provide insights in these fluctuations in patients’ everyday lives beyond our existing insight delivered by the snapshots of classic study designs.

First, mHealth thus offers opportunities to answer research questions that were difficult to investigate before. For example, the ‘Cloudy with a chance of pain’ study investigated the effect of weather parameters on pain and similar PROs.^90^ Before the use of mHealth, it would be near impossible to have a clear overview of a person`s specific whereabouts and the exact weather circumstances. Yet, now by coupling the weather forecast data together with a smartphone application that registers frequently a selection of PROs of patients with chronic pain, together with the GPS location of the phone, such endeavours can be conducted. Although the study found a relationship between pain and humidity, the authors were confronted with analytical issues and had to make various assumptions to tackle inter- and intra-participants differences. Hence, mHealth is a study design type that collects the data necessary to find new insights, yet methods for identifying clear-cut answers need further investigation.

Second, mHealth seems to allow recruitment of large patient populations in a limited amount of time. Recruitment to ‘Cloudy with a chance of pain’ for example went ballistic when the senior investigator appeared on a morning news television broadcast. A few tens of thousands of participants were recruited. This recruitment speed was a great success, however considering the adherence to the mHealth app, the enthusiasm to contribute by using the tool regularly among participants was limited since only 25% opened the app once.^91^ Perhaps it is better to think in terms of adherence to the use of mHealth compared to merely inclusion in the study. Thresholds to download an app can be low, yet it is more challenging to maintain the adherence. A few principles are used in many mHealth studies such as push notifications or feedback to the participant, yet success rate is not always clear, although mHealth user appreciate these methods.^92,93^ Some avenues that deserve more attention make use of gamification principles to increase user engagement or foster certain health behaviours.^94^ Such persuasive values require specific design techniques including offering praise, providing reminders, and emulating social agents or support. By using persuasive principles such as gamification, mHealth tools can change or shape attitudes or behaviours or both without using pressure or deception.^95^

In sum, mHealth is useful both in practice and in research, but comes with more limitations than may be initially anticipated. MHealth is being employed in various disciplines^96^ and procedures of different initiatives can also offer possibilities for patients with RMDs how to use mHealth optimally. However, creating mHealth applications for rheumatology purposes should take the opinion of every stakeholder into account,^92^ and the final mHealth product should be a result of a standardized process. These prerequisites seem intuitive, yet many mHealth apps in rheumatology do not seem to adhere to many of these basic principles.

### Synthesis and integration in the rheumatology clinic 2030

Many technologies have advanced over the past decade that might further flourish to impact clinical practice in the 2030s. Mobile health applications, electronic medical records, biobanking and integrative analyses by artificial intelligence seem to be the forerunners that will facilitate rheumatology health care professionals in a better, personalized manner to provide optimal patient care. Table 1 lists the opportunities and challenges for these topics accompanied by key references. The implementation of mHealth and digitization of health care is heterogenous across the globe and likewise the perspectives and willingness for change.^97,98^ This consequently implies that certain areas will be more advanced than others, independently of the general notion that e-health is a driver of health equity. Already some years back a patient and rheumatology researcher initiative started one of the most integrative mhealth apps /registries in the United States. The Arthritis Action app allows reporting, sharing PROs, medication data, but also importing EHR data. The patient is in the centre of managing, controlling as well as consenting to, what data to be shared for further research purposes.^99^ In many clinics around the world transition in healthcare delivery has already started before the pandemic. However, the COVID19 pandemic further revolutionized routine care by prompting many rheumatologists and patients to rely on telemedical aids, from simple phone calls to video conferences, but also e-prescriptions and referrals.^100,101^ Some studies have already shown that telemedical appointments may replace certain types of physical visits (e.g. follow-up visits) leading to non-inferior outcomes^102–104^ Ideally future hybrid management of care can be prepared with patients beforehand, using examples like guides for musculoskeletal examination for telemedicine.^105^ All these advancements might need standard frameworks and regulations as well as manuals for rheumatology practitioners to act in a legal, best-practice, and financially reimbursed space.^106^ In particular for mhealth tools like apps for monitoring of symptoms and impact of disease clinics and investigators have tested different concepts for use in clinical care and connection to their registers. App-based assessment is similarly accepted by patients as completing PRO measures in the clinic, a discussion about the patient scores on PROs may even improve the patient-physician relationship.^107–109^

**Figure 1.**
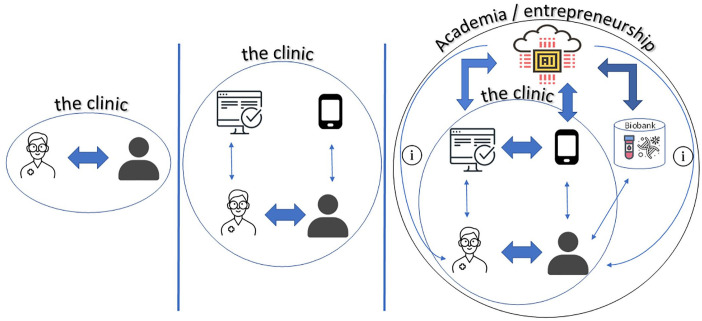
Previous, current and future clinical care in rheumatology. In the past, clinical practice focused only on patient-physician interaction. Nowadays, electronic health records and mHealth applications deliver extra data towards this interaction. In the future, we foresee additional information pathways such as biobank data including ‘omics’ data. Artificial Intelligence (AI) Algorithms will be developed to help integrate these data streams for the benefits of patient-physician interaction and patient outcomes.

Our eventual goal, as a rheumatology community, would be make healthcare more effective, efficient, and tailored to an individual patient. The ideal strategy and implementation for the processing of EHR, registries and passive and active monitoring data of people with or at-risk for RMDs will still take some considerable time but might likely be in place in some countries in 2030. Figure 1 illustrates an idea of this evolution of clinical care in rheumatology.

The need for better outcome prediction models is evident.^110^ A recent paper described a machine learning approach for flare prediction within a RCT tapering trial.^74^ Whether this approach will effectively work in large real-world datasets is unclear. Collaboration and multidisciplinary teamwork of medical experts, biostatisticians, and data experts is crucial for developing systems tailored to the needs of health care professionals and systems, prioritizing useful, and sensible thresholds.^111^ Artificial intelligence is the key that would facilitate a real-time clinical decision support system based on all available data. Other disciplines have trialled different decision support systems based on various technical models concerning medication side effect, need for further investigations but mostly risk assessment (e.g. sepsis, thromboembolisms, anticoagulation).^112–114^ These implementations and systems need to be accompanied by educational and evaluation measures to avoid an increase of work burden for healthcare professionals and even to avoid fatigue and bore-out of staff being forced to respond to an array of useless alerts. In the vast majority of cases of customized clinical decision support systems, their usage has led to improved outcomes.^112,114^ Building the technical and modelling infrastructure to use and integrate different data sources by means of machine learning to improve accuracy of management decisions will continue to develop to scalable approaches as long as a regulatory and ethical framework is set. A major challenge is to combine different data sources because of legal and data-security aspects. The creation of the European health data space (EHDS) by the European Commission aims to create a legal framework, which defines secondary use of data for all EU member states. The EHDS should harmonize legislations, standardize and certify the use of health data by different stakeholders and invests in capacity building of (e)health literacy of people. By these means, a trusted data governance should align, to tackle questions of improved patient safety, treatment strategies, and prevention.^115,116^ Forerunner projects that allow for interactive exchange between patient data reporting and monitoring, providing GDPR compliant access and editing options from patient and provider side, like in the PICASO project point in stronger patient empowered future.^117^

In sum, we live in an era of Big Data coming from various sources such as electronic healthcare records, mHealth applications and biobank omics data (Table 2). At the same time, methodologies such as artificial intelligence enable Big Data analysis and provide hope for continuous optimization of clinical care. However, many challenges still lay ahead before these steppingstones in research will make their way into the clinic. We look with an optimistic view into the next decade, that the bandwidth of technological advancements finds an integrative solution for implementation.

**Table 2.** Take-home messages and key references per topic.

TOPIC	MUST READ
**Electronic health records (EHRs)**	
EHRs offer potential to answer ambitious clinical and/or research questions through analyses of large amounts of data.	Pfeiffer *et al.*^29^
The use of EHR in rheumatology is in its early stages. Machine learning techniques to identify RMD diagnoses in EHR are just being developed.	Maarseveen *et al.*^34^
**Biobanks for clinical care**	
Combining clinical with data of multiple “omics” including genomics, proteomics, transcriptomics, epigenomics, and microbiomes is key to improve our knowledge of RMDs.	Winthrop *et al.*^35^
Biobanks are progressively used to address vital public health questions, yet the connection between biobanking with clinical care is still limited.	Coppola *et al.*^38^
**Artificial intelligence (AI) to support clinical care**	
AI methods help advance our understanding of pathogenesis, risk stratification and outcome prediction, and inform novel research avenues for identification of new drug targets and options for drug repurposing in rare diseases.	Kingsmore *et al.*^50^
AI methods are still rudimentary due to small sample size, lack of external validation, and implementation challenges in various clinical data sets.	Kedra *et al.*^77^
**Remote monitoring**	
The ubiquity of consumer smartphones and smartwatches provides opportunities to collect large amounts of both self-report and passively measured data.	Austin *et al.*^84^
A vast heterogeneity is present in designs, purposes and users of self-management mHealth apps in RMDs. Challenges are app relevance, involvement of stakeholders in the design process and high turnover rates.	Najm *et al.*^89^
Although mHealth is used mostly for research purposes, examples exist how it could provide insights in fluctuations in patients’ everyday lives beyond our existing insight.	Shaw *et al.*^108^
**The rheumatology clinic 2030**	
Mobile health applications, electronic medical records, biobanking and integrative analyses by artificial intelligence seem to be the forerunners that will facilitate precision medicine by healthcare professionals.	Richter *et al.*^117^
